# Size limits and fission channels of doubly charged noble gas clusters†

**DOI:** 10.1039/d4cp00658e

**Published:** 2024-03-26

**Authors:** Ianessa Stromberg, Stefan Bergmeister, Lisa Ganner, Fabio Zappa, Paul Scheier, Olof Echt, Elisabeth Gruber

**Affiliations:** a Institut für Ionenphysik und Angewandte Physik, Universität Innsbruck Innsbruck Austria e.gruber@uibk.ac.at olof.echt@unh.edu; b School of Chemistry, University of Edinburgh Edinburgh UK; c Department of Physics, University of New Hampshire Durham USA

## Abstract

Small, highly charged liquid droplets are unstable with respect to spontaneous charge separation when their size drops below the Rayleigh limit or, in other words, their fissility parameter *X* exceeds the value 1. The absence of small doubly charged atomic cluster ions in mass spectra below an element-specific appearance size *n*_a_ has sometimes been attributed to the onset of barrierless fission at *X* = 1. However, more realistic models suggest that *n*_a_ marks the size below which the rate of fission surpasses that of competing dissociative channels, and the Rayleigh limit of doubly charged van der Waals clusters has remained unchartered. Here we explore a novel approach to form small dicationic clusters, namely by Penning ionization of singly charged noble gas (Ng) clusters that are embedded in helium nanodroplets; the dications are then gently extracted from the nanodroplets by low-energy collisions with helium gas. We observe Ng_*n*_^2+^ ions that are about 40% smaller than previously reported for xenon and krypton and about 20% for argon. These findings suggest that fission barriers have been underestimated in previous theoretical work. Furthermore, we measure the size distributions of fragment ions that are produced by collisional excitation of mass-selected dications. At lowest collision gas pressure, dicationic Kr and Xe clusters that are smaller than previously observed are found to evaporate an atom before they undergo highly symmetric fission. The distribution of fragments resulting from fission of small dicationic Ar clusters is bimodal.

## Abstract 2

Sequential ionization of helium nanodroplets doped with noble gases produces doubly charged clusters that are much smaller than observed previously.

## Graphical abstract

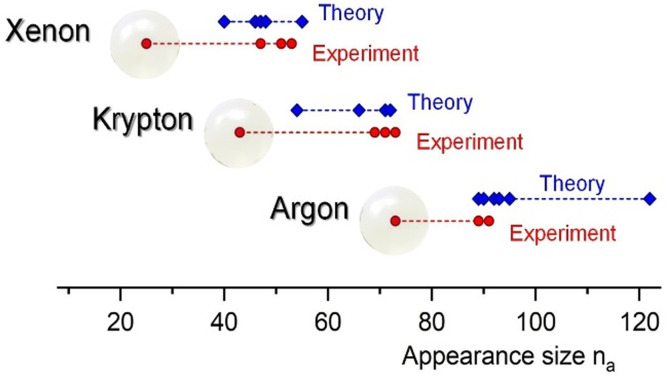

## 1 Introduction

The long-range Coulomb repulsion between like charges renders highly charged many-body systems such as atomic nuclei, atomic clusters, or macroscopic objects prone to charge separation.^1–3^ As shown by Lord Rayleigh, a spherical, uniformly charged droplet becomes unstable with respect to any small quadrupolar deformation when its Coulomb energy *E*_Coulomb_ exceeds twice its surface energy *E*_surface_, *i.e.* when the fissility parameter *X* = 1/2*E*_Coulomb_/*E*_surface_ exceeds a value of 1.^1,4^ One customarily refers to this regime, in which the system exhibits multifragmentation, as “Coulomb explosion”.^5–9^ Atomic clusters with *X* > 1 can be prepared, for example, by exposing them to intense, very short laser fields,^10–12^ by collisions with highly charged atoms,^13,14^ or by core level excitation of an atom in the cluster.^8,15^

Charge separation in the regime with *X* < 1 is usually referred to as fission.^5,6,8^ The fission barrier which separates the bound initial system from the fission products has to be overcome by excitation or, for atomic nuclei, be penetrated by tunneling. There are several ways in which atomic clusters with *X* < 1 may be formed; they may originate from multiple ionization of large neutral clusters, or they may be the products of Coulomb explosion. Either way, they will be thermally excited. Questions that are often addressed in the fission regime are: (i) what is the Rayleigh limit, *i.e.* the size *n*_Ray_ of a *z*-fold charged cluster A_*n*_^*z*+^ at which the fission barrier vanishes? (ii) what is the smallest experimentally observable size of A_*n*_^*z*+^? Obviously, this so-called appearance size *n*_a_ is limited by *n*_a_ > *n*_Ray_. (iii) What happens if a cluster A_*n*_^*z*+^ undergoes unimolecular or collision-induced fragmentation? Will it eject neutral monomers or will it fission? If so, what is the size distribution of the fission products?

These and many other questions have been pursued for multiply charged clusters composed of metal and noble gas (Ng) atoms, inorganic and organic molecules, and fullerenes.^4,11,16–19^ Here we will focus our attention on homonuclear noble gas clusters; other systems have their own idiosyncrasies. Double-ionization of benzene clusters, for example, can lead to the formation of inter-ring covalent C–C bonds which substantially reduce the Coulomb repulsion.^20^ Doubly charged heteronuclear clusters, and even dimers such as NeXe^2+^ and HeCa^2+^, may be thermodynamically stable with respect to charge separation because the two charges reside on the same atom; charge transfer from the dication to the neutral ligand would be endothermic.^21–26^ We will also ignore multiply charged cluster anions where electron detachment represents an additional relaxation channel.^27–29^

That restricts our discussion to Ng_*n*_^*z*+^ with Ng = He, Ne, Ar, Kr, Xe. The smallest reported appearance sizes of dications are *n*_a_ = 1.00 × 10^5^ for He,^30^ 284 for Ne,^31^ 91 for Ar,^32–34^ 69 for Kr,^35^ and 47 for Xe.^36,37^ These values have been obtained by electron ionization of neutral clusters combined with mass spectrometry. The increase of the ion yield of dications above *n*_a_ is, in general, very steep. Slightly larger appearance sizes reported for Kr and Xe in earlier reports are due to lower mass resolution, ion yield or signal-to-noise ratio.^16,38,39^

The absence of doubly charged clusters below *n*_a_ suggests that their lifetime with respect to fission is much less than the time required for their detection (which is typically of the order 10^−4^ s). The fission barrier *E*_bar_ will rapidly decrease with decreasing cluster size while the evaporation energy *E*_vap_ is approximately constant. Thus, it seems reasonable to assume that *n*_a_ marks the size where the rate of fission exceeds that of evaporation or, roughly, *E*_bar_ ≈ *E*_vap_. This approximation has found experimental and theoretical support.^4,34,40–45^ Surprisingly, though, the dominant product of unimolecular dissociation of doubly charged Ar and CO_2_ clusters near their appearance size are slightly smaller dications; no fission products are observed.^33,46^ Molecular dynamics simulations^47,48^ and the deformable liquid drop model^41^ hint at a solution to this conundrum: charge separation in a nascent dication, produced from a neutral, approximately spherical cluster, excites the collective stretching mode which will overcome the fission barrier within one vibrational period (less than about 100 ps) if its energy exceeds the fission barrier. Else, fission has to be driven by thermal energy which is abundant, thanks to the energy released upon formation of dimeric or trimeric ion cores, but which is much less efficient in overcoming the fission barrier. Thus, after some 100 ps the balance between the two competing dissociation channels shifts in favor of evaporation.

The excitation of the stretching mode is inherent to molecular dynamics studies of doubly charged noble gas clusters that are vertically ionized,^6,47–50^ and the calculated appearance sizes agree with reported experimental appearance sizes within 10%. Alternatively, appearance sizes are computed within the spherical liquid drop model.^16,19,51,52^ In this approach the crude contacting-sphere approximation is usually adopted and one merely computes the size where the energies of the precursor and the products are equal. Even so, good agreement with experimental appearance sizes is obtained for most systems.

The happy agreement between experiment and theory has pushed the question of the Rayleigh limit to the backstage. At what size does the fission barrier vanish for a doubly charged noble gas cluster? A rule of thumb emerging from past work on metal clusters is that the fissility of the smallest observed doubly charged clusters (*i.e.* the ratio *n*_Ray_/*n*_a_) is about 0.3 to 0.4.^4,13^ Recently we have succeeded to form doubly charged clusters of alkali metals and of coronene that were much smaller than previously observed by embedding the neutral precursors in helium nanodroplets (HNDs).^53,54^ Similar experiments with CO_2_ clusters, on the other hand, did not lead to an appreciable decrease of the appearance size.^55^

**Fig. 1:**
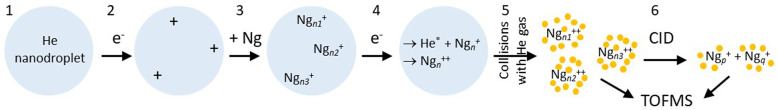
Synthesis of doubly charged clusters by electron ionization of helium nanodroplets (HNDs) followed by doping with noble gas (Ng) atoms and 2nd electron ionization. Dopant ions are extracted from the HNDs by collisions with He gas and analyzed in a time-of-flight mass spectrometer (TOFMS). Step 6 (collision-induced dissociation (CID) of mass-selected ions) is optional.

Here we apply a novel approach to form small multiply charged clusters. Fig. 1 illustrates the method. First, large helium nanodroplets (HNDs) are prepared by supersonic expansion of He and ionized by electrons, resulting in highly charged droplets.^30^ These are doped with noble gas atoms which will nucleate at the multiple charge centers, leading to singly charged Ng clusters embedded in the HND. Subsequent bombardment of the droplets with electrons converts the embedded Ng_*n*_^+^ to dications, presumably by Penning ionization.^56,57^ The dications are then gently extracted by low-energy collisions of the droplets with helium gas, leading to bare (or nearly bare) Ng_*n*_^2+^.

Using this novel approach, the appearance size of Xe_*n*_^2+^ is reduced by a factor 2 to *n*_a_ = 25.^58^ This value is even smaller than the Rayleigh limit computed by Casero *et al.* with the deformable droplet model.^41^ If we estimate *n*_Ray_ from the Rayleigh formula using the surface tension of liquid Xe and taking the effect of the droplet size into account,^60,61^ we find that the fissility of Xe_25_^2+^ is *X* = 0.84. The appearance sizes of Kr_*n*_^2+^ and Ar_*n*_^2+^ are strongly reduced as well.

Furthermore, we have measured the dissociation channels of collisionally excited doubly charged cluster ions. For the smallest precursor clusters, with sizes approximately 10% below previously reported appearance sizes, the size distributions of fission fragments measured at the lowest collision pressure is very symmetric, especially for Kr and Xe. For these systems, fission appears to be preceded by evaporation of an atom, and accompanied by evaporation of no more than one additional atom. Collisional excitation of larger dicationic precursors leads to evaporation of several atoms; the product ions do not fission until their size has dropped well below previously reported appearance sizes.

The high resolution of the mass spectrometer (10 000) makes it possible to identify Ng_*n*_^2+^ cluster ions that have the same size-to-charge ratio *n*/*z* as singly charged clusters. This helps, for example, to distinguish between loss of an atom from Xe_43_^2+^ and fission into Xe_21_^+^. We also identify anomalies in the abundance distributions of doubly charged Kr and Xe clusters; they may reflect anomalies in the size dependence of the fission barrier, caused by geometric shell effects.^51^

## 2 Experimental

An earlier version of the experimental apparatus, described in ref. 62 and 63 was recently upgraded by adding a second electron impact ionizer after the pickup cell.^64^ A schematic of the system is presented in the ESI† (Fig. S1).

Experiments are run in one of two modes. In one of them, products from collision-induced dissociation (CID) of mass-selected cluster ions (step #6 in Fig. 1) are analyzed in a time-of-flight mass spectrometer (TOFMS). In the other mode, step # 6 is omitted, and the TOFMS probes the complete distribution of cluster ions that emerge after stripping He from the dopant ions. With reference to Fig. 1, details are as follows:

1. Helium nanodroplets are produced in an expansion of cold helium gas (pressure 22 bar) through a 5 μm nozzle (temperature 8.5 K) into vacuum; the HNDs contain of the order of *N* ≈ 10^7^ atoms.^65^

2. The droplets are ionized by an intense electron beam at 40 eV and an emission current of about 100 μA. The resulting highly charged He_*N*_^*z*+^ droplets will contain a few to a few dozen positive charges that are located on He_3_^+^ ions.^30,66,67^ The droplets then pass through an electrostatic deflector which selects ions by their mass-to-charge ratio, hence their size-to-charge ratio *N*/*z*. In the present work the deflector was tuned to *N*/*z* ≈ 1.5 × 10^5^.

3. The ions are guided through a cell filled with low-density gas where they collide with and pick up noble gas (Ng) atoms. The dopants agglomerate inside the helium droplets at charge centers to form *z* singly charged clusters Ng_*n*_^+^, where *n* and *z* follow some broad distribution.

4. The beam of doped droplets is crossed by another intense electron beam at about 40 eV. The electrons may directly ionize the singly charged dopant ions but, more likely, the dopant ions are converted to dications by an intracluster Penning mechanism,^56,57,67^ as discussed in Section 4.

5. The doped droplets collide with helium gas in a radio frequency hexapole ion guide, leading to the gentle escape of the dopant ions from the droplet. Further downstream, the resulting ions are perpendicularly extracted by a pulsed electric field into a TOFMS equipped with an electrostatic reflectron which measures their mass-to-charge ratio *m*/*z*. The mass resolution is about 10^4^ FWHM (full-width at half-maximum).

6. In an optional step, a quadrupole mass filter is used to select cluster ions with a specific mass-to-charge ratio. The selected precursor ions are accelerated and sent through a hexapole ion guide (length 182 mm) where they collide with a thermal gas of argon; product ions are analyzed in the TOFMS. The kinetic energy of the cluster ions passing through the guide is 2 eV or larger. The residual gas pressure in the ion guide is about 5 × 10^−7^ mbar; it consists of mostly helium which leaks from the evaporation cell and, in experiments with Ar clusters, argon leaking from the pickup cell. The pressure sensor is mounted externally; the pressure that is read when argon is admitted into the collision cell will underestimate the actual Ar pressure by a large factor.

## 3 Results

### 3.1 Mass spectra

#### 3.1.1 Krypton and xenon

**Fig. 2:**
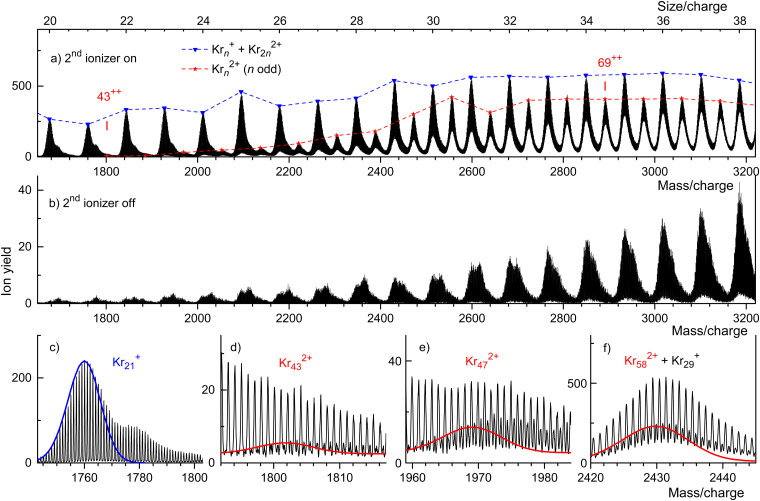
Mass spectra of Kr cluster ions. Triangles and asterisks in panel a flag cluster ions for which the size-to-charge ratio *n*/*z* (indicated along the upper abscissa) is integer and half-integer, respectively. Kr_43_^2+^ is the smallest odd-numbered dicationic cluster. Panel b: no dications appear when the second ionizer is turned off. Four sections of the spectrum in panel a are reproduced in panels c through f. Lines indicate the expected distributions of isotopologues of Kr_21_^+^, Kr_43_^2+^, Kr_47_^2+^, and Kr_58_^2+^.

Mass spectra of HNDs doped with Kr are presented in Fig. 2. In Fig. 2(a), recorded with both ionizers turned on, we see a prominent series of mass peaks positioned at integer multiples of *m*/*z* = 83.8, the atomic weight of Kr. The mass peaks are flagged by triangles connected by a dashed line. These mass peaks are due to Kr_*n*_^+^ but they may also contain contributions from Kr_2*n*_^2+^ (recall that mass spectrometers measure the mass-to-charge ratio *m*/*z*, hence the size-to-charge ratio *n*/*z* of Kr_*n*_^*z*+^).

A weaker series of mass peaks appears exactly midway between the Kr_*n*_^+^ series. These ions are due to doubly charged clusters Kr_*n*_^2+^ containing an odd number of atoms. The appearance size of dications, *n*_a_ = 43, is indicated in Fig. 2(a). Also indicated is the much larger appearance size, *n*_a_ = 69, reported previously.^35^

The spectrum in Fig. 2(b) was recorded under the same conditions as the one in Fig. 2(a) but with the second ionizer being turned off. As expected, no doubly charged clusters are formed in that mode.

The mass peaks in Fig. 2(a) and (b) appear to be broad even though the mass resolution is about 10 000. Krypton has six naturally occurring isotopes ranging from ^78^Kr to ^86^Kr; a cluster ion containing dozens of Kr atoms will feature many different isotopologues. At high resolution, when individual isotopologues are resolved, each mass peak assigned to a specific cluster Kr_*n*_^*z*+^ comprises dozens of sub-peaks that merge into a black band in the graph.

This is demonstrated in Fig. 2(c) which zooms into Fig. 2(a) in the mass region of Kr_21_^+^. Subpeaks are, as expected for monocations, separated by Δ*m*/*z* = 1. The expected distribution of isotopologues calculated from the natural abundance of Kr isotopes is indicated by the blue line; it matches the experimental data up to *m*/*z* ≈ 1765.^35,68,69^ The excess ion yield observed for larger *m*/*z* values is due to Kr_21_^+^ complexed with one or more He atoms; the ions may also contain a water molecule.

These mixed ions overwhelm the Kr_43_^2+^ signal as shown in Fig. 2(d). However, for dications the spacing between different isotopologues is Δ*m*/*z* = 0.5. Therefore, isotopologues of Kr_43_^2+^ whose mass number is odd appear midway between mass peaks due to isotopologues of He_*p*_Kr_21_^+^ and He_*p*_H_2_OKr_21_^+^ (*p* = 0, 1, 2…). The expected distribution of Kr_43_^2+^ isotopologues is indicated by the red line in Fig. 2(d), scaled to match the onset of the experimental data. The excess yield of dications seen in the right half of Fig. 2(d) is due to He_*p*_Kr_43_^2+^.

The yield of Kr_*n*_^2+^ clusters rapidly increases with size *n* beyond the appearance size *n*_a_ = 43; this is illustrated for Kr_47_^2+^ in Fig. 2(e). Additional data are shown in the ESI† (Fig. S2). Around *n* ≈ 60, the yield of dications is about twice that of monocations with the same *n*/*z* value, also see Fig. S6 in the ESI.† Note, however, that the ratio of ion yields will depend on various experimental parameters which were deliberately tuned to optimize the yield of dications. Furthermore, the detection efficiency of dications might exceed that of monocations with the same *n*/*z* value.

Note that contributions of singly and doubly charged clusters to a mass peak with an integer value of *n*/*z* can be determined separately if the mass resolution exceeds Δ*m*/*z* = 0.5. This is demonstrated in Fig. 2(f) for the mass peak at *n*/*z* = 29.

**Fig. 3:**
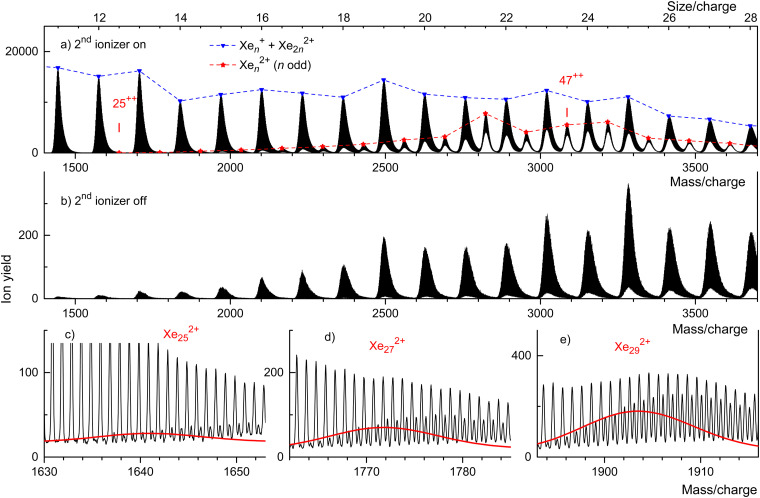
Mass spectra of Xe cluster ions. Triangles and asterisks in panel a flag cluster ions for which the size-to-charge ratio *n*/*z* is integer and half-integer, respectively. Xe_25_^2+^ is the smallest odd-numbered dicationic cluster. Panel b: no dications appear when the second ionizer is turned off. Panel c: three sections of the spectrum shown in panel a. Lines indicate the expected distribution of isotopologues of Xe_25_^2+^, Xe_27_^2+^, and Xe_29_^2+^.

Fig. 3 displays our results for HNDs doped with Xe atoms; they resemble those for Kr doped HNDs. Xe (atomic weight 131.29) has 9 naturally occurring isotopes; 7 of them (^128^Xe through ^136^Xe) have abundances exceeding 1%. Hence the mass peaks of Xe cluster ions seen in Fig. 3(a) are broad; they are due to Xe_*n*_^*z*+^ with integer and half-integer values of *n*/*z*. The appearance size of dications is *n*_a_ = 25, much smaller than the previously reported value *n*_a_ = 47.^36^ No dications are observed when the 2nd ionizer is turned off (Fig. 3(b)).

Isotopologues of Xe_*n*_^2+^ with odd mass numbers are seen in Fig. 3(c) through Fig. 3(e) for *n* = 25, 27, and 29, respectively. The expected distributions of isotopologues, indicated by the red lines, level off more quickly than the experimental data because of contributions from He_*p*_Xe_*n*_^2+^.

#### 3.1.2 Argon

**Fig. 4:**
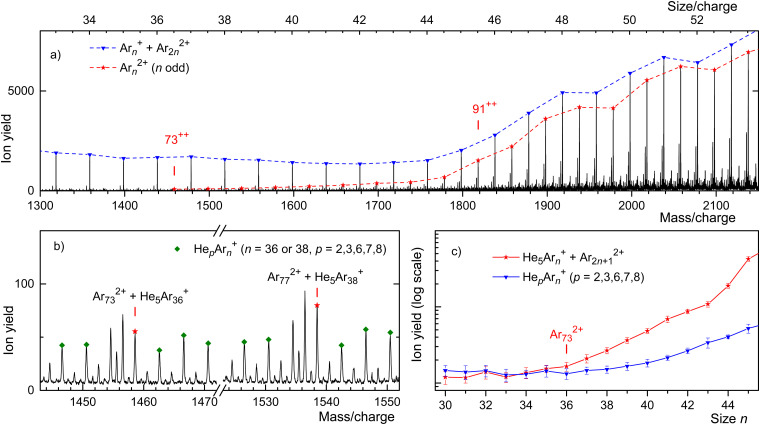
Panel a: a mass spectrum of Ar cluster ions. Asterisks and triangles flag cluster ions for which the size-to-charge ratio *n*/*z* is half-integer and integer, respectively. Ar_73_^2+^ is the smallest odd-numbered dicationic cluster. Panel b zooms into two regions of the spectrum. Mass peaks flagged by diamonds are due to Ar_36_^+^ or Ar_38_^+^ complexed with 2, 3, 6, 7, or 8 He atoms. Mass peaks due to He_5_Ar_36_^+^ and He_5_Ar_38_^+^ are marked by asterisks; they coincide with mass peaks due to bare Ar_73_^2+^ and Ar_77_^2+^, respectively. The data in panel c suggest that the appearance size of dicationic argon clusters is *n*_a_ = 73.

Ar has one dominant isotope, ^40^Ar, with an abundance of 99.60%. Its other isotopes, ^36^Ar and ^38^Ar, have abundances of 0.34 and 0.06%, respectively. The mass spectrum of Ar clusters displayed in Fig. 4(a) shows a prominent series of mass peaks (marked by triangles) due to ^40^Ar_*n*_^+^ which may include contributions from even-numbered ^40^Ar_*n*_^2+^. Odd-numbered ^40^Ar_*n*_^2+^ (marked by asterisks) appear midway between the monocations. Their yield is small but it rises quickly beyond *n*/*z* ≈ 44 where their peak heights quickly approach those of ^40^Ar_*n*_^*z*+^ with integer values of *n*/*z*. Assuming that the ion yield of dicationic clusters does not oscillate with size *n*, one has to conclude that the mass peaks with integer values of *n*/*z* are mostly due to dicationic rather than monocationic clusters beyond *n*/*z* ≈ 44.

This assertion can be substantiated by analyzing the isotope pattern. In the size range discussed here, the second-most abundant isotopologue is due to ^36^Ar^40^Ar_*n*−1_. The corresponding mass peak is separated from the main isotopologue by Δ*m*/*z* = 4 for monocations but by Δ*m*/*z* = 2 for dications. As shown in detail in the ESI† (Fig. S3), dications form the dominant contribution for *n*/*z* ≥ 49, while mono- and dications contribute about equally for *n*/*z* = 45.

Unfortunately, for smaller values of *n*/*z* the isotopic analysis becomes inconclusive because of contributions from ions other than bare Ar clusters. Identifying mass peaks due to odd-numbered dications faces two challenges. First, all Ar isotopes have even mass numbers. Hence, doubly charged Ar clusters do not show the Δ*m*/*z* = 0.5 spacing between isotopologues that was characteristic of Kr and Xe clusters. Second, the *m*/*z* values of He_5_^+^ and ^40^Ar^2+^ differ by only 0.032. The mass resolution required to resolve He_5_Ar_*n*_^+^ and Ar_2*n*+1_^2+^ exceeds the resolution of our instrument in the size range of interest by a factor 5.

Two sections of the Ar mass spectrum displayed in Fig. 4(b) demonstrate the problem. Mass peaks due to He_*p*_^40^Ar_*n*_^+^ with *n* = 36 or 38 and *p* = 2, 3, 6, 7, 8 are marked by diamonds (the peak for *p* = 4 has been omitted because a water contamination affects the data). The mass peaks for *p* = 5 are marked by asterisks; they include contributions from ^40^Ar_2*n*+1_^2+^.

In order to pinpoint the appearance size of dications, we plot the yield He_*p*_^40^Ar_*n*_^+^, extracted with the software package IsotopeFit^70^ and averaged over *p* = 2, 3, 6, 7, 8, *versus n* in Fig. 4(c) (blue triangles). The error bars reflect the standard deviation of the average. The yield of the mass peak due to (He_5_^40^Ar_*n*_^+^ + ^40^Ar_2*n*+1_^2+^) is plotted with red asterisks; the error bar is reported by the software. Beginning at about *n* = 73, the yield of the mass peak that includes contributions from doubly charged clusters grows much faster with increasing *n* than the yield of He_*p*_^40^Ar_*n*_^+^ averaged over *p*. We conclude that *n*_a_ = 73 is the appearance size of Ar_*n*_^2+^. The smallest dicationic Ar cluster observed previously was Ar_91_^2+^.^32–34^

The appearance sizes *n*_a_ obtained in the present work are collected in Table 1 together with previously reported appearance sizes. Data for neon are included for completeness. Theoretical “appearance sizes” are listed as well, but they do not always refer to one and the same quantity; this will be discussed in Section 4.

**Table 1:** Appearance sizes *n*_a_ of doubly charged noble gas clusters (columns 2, 4, and 5). The smallest dications observed in CID spectra are listed in column 3, with precursor sizes in parenthesis. Column 6 lists Rayleigh limits computed from eqn (3) for surface tensions pertaining to solid and liquid droplets and, for Xe, computed from a deformable liquid drop model^41^

Element	*n*_a_ (this work)	CID (this work)	Literature (exp)	Literature (theory)	*n*_Ray_
Ne			284^a^	278^i^, 465^j^, 632^k^, 635^l^, 868^e^	
Ar	73	93 (115)	91^b^^c^^e^	89^i^, 90^m^, 92^l^^n^, 93^k^, 95^o^^r^, 122^e^	39_sol_, 56_liq_
Kr	43	58 (61), 65 (77)	69^d^, 71^e^, 73^f^	54^l^, 66^i^, 71^e^, 72^m^	23_sol_, 32_liq_
Xe	25	39 (43), 43 (49), 43 (55)	47^g^, 51^e^, 53^h^	36^l^, 46^e^, 47^i^, 48^p^, 49^r^, 55^m^^o^^q^	15_sol_, 21_liq_, 30^r^

a Ref. 31.

b Ref. 34.

c Ref. 32.

d Ref. 35.

e Ref. 16.

f Ref. 72.

g Ref. 36.

h Ref. 39.

i Ref. 52.

j Ref. 73.

k Ref. 74.

l Ref. 51.

m Ref. 50.

n Ref. 48.

o Ref. 75.

p Ref. 49.

q Ref. 47.

r Ref. 41.

The appearance sizes for Kr and Xe clusters obtained here are about 40% lower than the lowest previously reported values; for Ar they are about 20% lower.

The mass spectra of Ar-doped HNDs show the appearance of triply charged clusters. However, their abundance is very small compared to that of various other ions with similar *m*/*z* value. Our best estimate for the appearance size of Ar_*n*_^3+^ is *n*_a_ ≈ 214, only slightly smaller than the value *n*_a_ = 226 reported previously.^71^ We will not discuss tricationic clusters any further.

### 3.2 Collision-induced dissociation

Ionization of van der Waals bound clusters is often accompanied by fragmentation. Conventional mass spectra do not reveal the precursors of the detected ions, but experiments in which precursor ions are mass-selected and subsequently excited can reveal the fragmentation channels. We have performed collision-induced dissociation (CID) experiments in which a long-lived ion is selected, excited by collisions with neutral atoms (in our experiment, argon), and the product ions are then analyzed in the TOFMS. For doubly charged clusters, obvious questions are: what is the preferred dissociation channel, evaporation (*i.e.* loss of neutral atoms) or fission? What is the smallest doubly charged fragment ion? If fission occurs, what is the size distribution of the fission fragments?

**Fig. 5:**
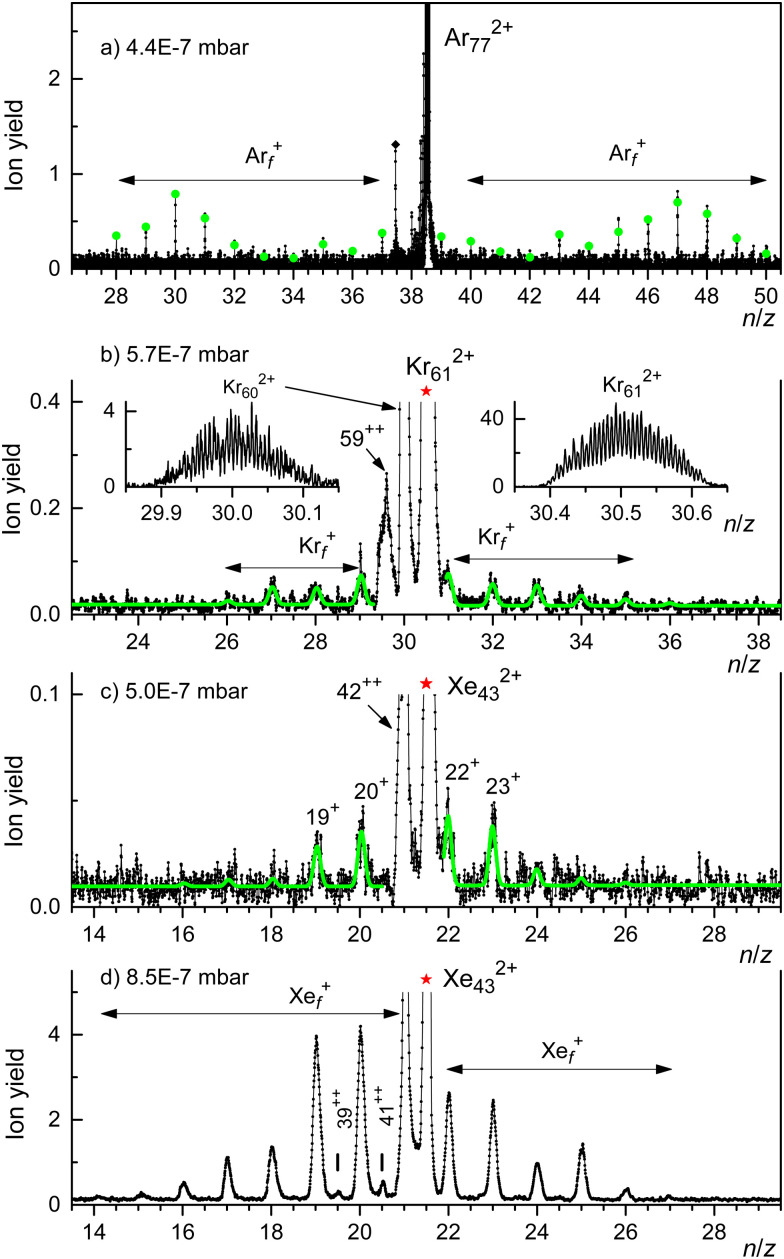
Panels a through c: fragment ions produced by collisional activation of Ar_77_^2+^, Kr_61_^2+^, and Xe_43_^2+^ with background gas. Most of the fragment ions are singly charged except for dicationic Kr and Xe clusters that result from evaporation of one or two atoms from the precursor. The spectrum in panel d reveals a strong increase in the yield of singly charged fragments and a shift of their size distribution to the left when the collision gas pressure is increased.

Spectra of collision-induced fragment ions are displayed in Fig. 5 for Ar_77_^2+^, Kr_61_^2+^, and Xe_43_^2+^ (panels a, b, c, respectively). These clusters are about 10% smaller than the previously reported appearance sizes. No argon was introduced into the collision cell. The background pressure was about 5 × 10^−7^ mbar (exact values are provided in the figure). We cannot tell to what extent unimolecular dissociation contributes to the observed fragment ion yield.

For Ar the mass filter was set to pass just one isotopologue, ^40^Ar_77_^2+^ (including, as discussed above, contributions from He_5_^40^Ar_38_^+^). For Kr and Xe the filter was set to pass all isotopologues that significantly contribute to clusters with the desired value of *n*/*z*.

For Ar_77_^2+^ (Fig. 5(a)) we observe fragment ion peaks at integer *n*/*z* values to the left and right of the precursor. The absence of mass peaks at half-integer *n*/*z* values indicates that the fragment ions are singly charged. At any rate, mass peaks to the right of the precursor must be fission fragments because *n*/*z* can increase only if *z* decreases. Conversely, the appearance of fragment ion peaks to the right of the precursor proves that its charge state was *z* > 1. (The mass peak marked by a diamond in Fig. 5(a), and other mass peaks immediately to the left of the precursor ion, may be due to fragments from ions other than Ar_77_^2+^ that passed through the mass filter).

The distribution of fission fragments in Fig. 5(a) is bimodal and nearly symmetric with respect to the precursor. The count rate in this measurement was very small (the ordinate indicates the number of events per bin), and the ion yield has been averaged over several adjacent bins. The spectrum was fitted with a set of equidistant Gaussians; the reported peak amplitudes are indicated by dots. The results of the fit are listed in Table 2, where *f*_−_ and *f*_+_ denote the weighted average size of fission fragments to the left and right of the precursor ions. The asymmetry parameter α of the distribution, defined as1
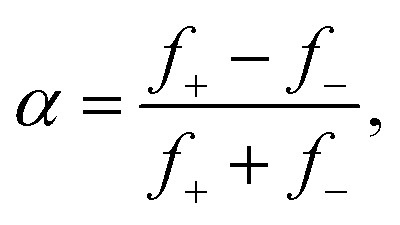
equals *α* = 0.17. The average number of neutral atoms emitted upon fission (*i.e.* the precursor size *n* − *f*_−_ − *f*_+_) equals 0.8.

**Table 2:** Average size of singly charged fragments that contain <50% and >50% of the doubly charged precursor ion (*f*_−_ and *f*_+_, respectively), extracted from the spectra in Fig. 5. Also listed are the asymmetry parameters α of the size distributions and the average numbers of lost monomers

Precursor	*f*_−_	*f*_+_	*α*	Monomer loss
Ar_77_^2+^	31.6	44.6	0.17	0.8
Kr_61_^2+^	28.1	32.4	≤0.07	≤0.5
Xe_43_^2+^	19.2	22.8	≤0.09	≤1.0

Most of the fragments from Kr_61_^2+^ and Xe_43_^2+^ (Fig. 5(b) and (c)) appear at integer *n*/*z* values, they are singly charged (exceptions are discussed below). Their size distributions are narrow and approximately symmetric with respect to the precursor ions. The spectra were fit by a set of equidistant Gaussians. The fitted curves are indicated by solid lines in the Figure; results are summarized in Table 2. The asymmetry parameters are even smaller than for fission of Ar_77_^2+^.

The data displayed in Fig. 5(b) and (c) were strongly smoothed by adjacent averaging; individual isotopologues are not resolved. However, the mass peak immediately to the left of the precursor ion had a much larger count rate. The spectra shown in the insets in Fig. 5(b) employ much weaker signal averaging; they reveal that the fragment at *n*/*z* = 30 is doubly charged, *i.e.* it is produced by evaporation of an atom from the Kr_61_^2+^ precursor. Evaporation of another atom produces Kr_59_^2+^. Evaporation of one atom is also observed for Xe_43_^2+^.

The prominence of doubly charged fragment ions that contain one atom less than the precursor ion renders our analysis of the size distribution of fission fragments incomplete for Kr and Xe. For Xe_43_^2+^, for example, we cannot determine the yield of Xe_21_^+^ fragment ions because their mass peak is buried under the Xe_42_^2+^ mass peak. If we could, the values of *f*_−_ and *f*_+_ would approach each other, and the asymmetry would be less. Furthermore, the spectrum in Fig. 5(c) suggests that the immediate precursor of the fission fragments is Xe_42_^2+^ rather than Xe_43_^2+^, which implies that the number of monomers lost upon fission is zero rather than 1. In short, the fragmentation of Xe_43_^2+^ is probably best characterized as

2a Xe_43_^2+^ → Xe_42_^2+^ + Xe

followed by

2b Xe_42_^2+^ → Xe_21−*q*_^+^ + Xe_21+*q*_^+^ with *q* = 0, 1, 2, and *α* < 0.09

When Ar is introduced into the collision cell, the yield of fragment ions increases strongly, and the fragment size distribution shifts to the left. Fig. 5(d) shows that fission of Xe_43_^2+^ produces singly charged fragments containing as many as 27 and as few as 13 atoms. We also see products of evaporation from Xe_43_^2+^, namely Xe_41_^2+^ and Xe_39_^2+^.

**Fig. 6:**
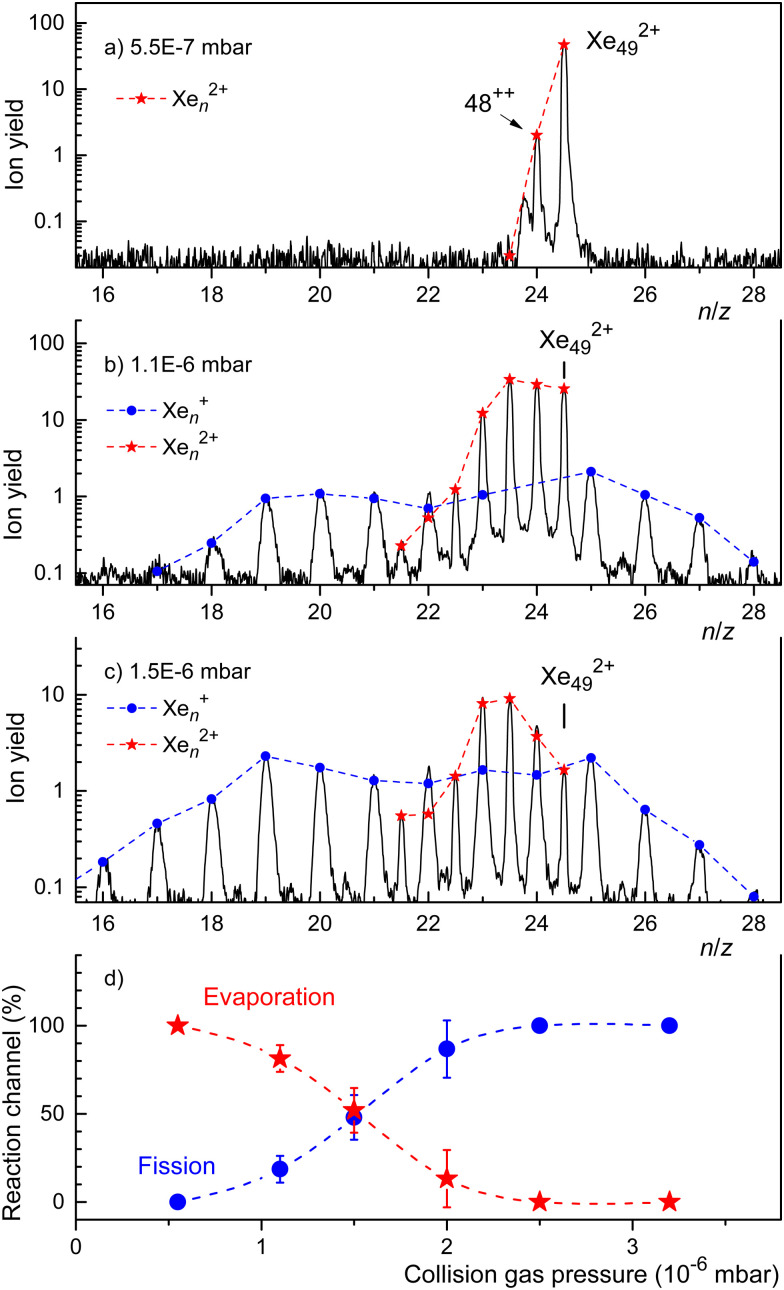
Panels a through c: fragment ions resulting from collisional excitation of Xe_49_^2+^ recorded with increasing collision gas pressure. Contributions from singly and doubly charged ions to the mass peaks are indicated by dots and asterisks, respectively. Panel d displays the relative yield of dications (evaporation) and monocations (fission) *versus* collision gas pressure.

In Fig. 5 we showed the results of CID experiments involving doubly charged clusters that were as small as practically possible, *i.e.* for which the ion yield was not too weak. In Fig. 6 we present CID results for a slightly larger cluster ion, Xe_49_^2+^. When no Ar gas is introduced into the collision cell we see only one reaction, evaporation of one atom. When the Ar pressure is increased, we see evaporation from the dication down to *n* = 43. Upon increasing the Ar pressure further to *P* = 1.5 × 10^−6^ mbar (Fig. 6(c)), the most notable change is a shift of the distribution of fission fragments to smaller clusters.

The spectra displayed in Fig. 6(a) through Fig. 6(c) involve strong signal averaging. Individual isotopologues are resolved with less smoothing, and for each mass peak we can determine the contribution of singly and doubly charged ions. These contributions are indicated in Fig. 6 by dots and asterisks, respectively. In Fig. 6(d) we plot the sum of all monocations (which result from fission) and dications (which result from evaporation) *versus* collision gas pressure. The relative yield of the former reaction channel increases with increasing collision gas pressure while the latter decreases. The probabilities are about equal if *P* ≈ 1.5 × 10^−6^ mbar.

The trend revealed by the data in Fig. 6(d) is supported by CID experiments with Xe_55_^2+^ that are displayed in the ESI† (Fig. S5). This precursor is larger than the previously reported appearance size (*n*_a_ = 47). For low Ar pressures the spectrum is dominated by dications resulting from evaporation. The yield of singly charged fragments remains weak until the collision pressure is high enough to produce dications near a lower limit of *n* = 43. For that pressure, the distribution of singly charged fragments reaches a maximum near *n* = 19. Evidently, the first effect of collisional excitation is to shrink the dication by evaporation; fission does not occur until some size limit is reached, where the dication fissions into two monocations of approximately equal size. These size limits, deduced from CID spectra of Xe_43_^2+^, Xe_49_^2+^, and Xe_55_^2+^, are compiled in Table 1. They slightly increase with the size of the precursor, from 39 to 43, but they are significantly smaller than the previously reported appearance size (*n*_a_ = 47).

CID experiments with Ar_115_^2+^ (see the ESI,† Fig. S4) support the notion that fission does not occur until evaporation has produced sufficiently small dications. Competition between fission and evaporation is restricted to a narrow size range. At low pressures, collisional excitation results in evaporation, producing dications down to *n* ≈ 93. Singly charged fission fragments are not formed until this size limit is reached.

CID spectra of Kr_*n*_^2+^ have been recorded for *n* = 61 and 77. Qualitatively, they confirm the conclusions drawn above for Xe and Ar clusters.

## 4 Discussion

Using a novel experimental approach, we have shown that doubly charged noble gas clusters much smaller than observed previously can be formed and detected in a high-resolution mass spectrometer.

In experimental work, the appearance size specifies the size of the smallest multiply charged cluster Ng_*n*_^*z*+^ that has been detected. Appearance sizes reported for doubly charged Ar, Kr, and Xe clusters have decreased over the years, but only slightly (see Table 1). One might assume that improvements in mass resolution, ion yield, and signal-to-background will lead to the detection of ever smaller dications, but one would be wrong. Eventually one will reach the Rayleigh limit where the fission barrier shrinks to zero, and fission of the nascent dication occurs within a fraction of a nanosecond.^47,48^ These ions would escape mass spectral detection which requires ion lifetimes of the order of 1 to 100 μs. In our instrument, ions must stay intact for roughly 50 μs in order to be detected.^76^

According to Rayleigh,^1^ a uniformly charged liquid droplet will become unstable with respect to quadrupolar deformation when its Coulomb energy exceeds twice its surface energy, or when the fissility parameter *X*, defined by the ratio of the two quantities, exceeds 1. This happens at the radius3
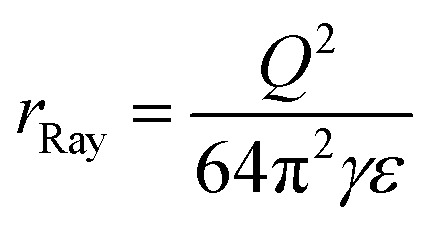
where *Q* is the charge, *γ* the surface tension, and *ε* the permittivity of the medium.

We will denote the cluster size at which Ng_*n*_^2+^ reaches the Rayleigh limit (*i.e.*, at which the fission barrier vanishes) by *n*_Ray_. The fissility factor *X* = *n*_Ray_/*n* will be a useful definition in the following discussion which will address the following topics:

• What is the significance of experimental appearance sizes?

• What is the significance of theoretical appearance sizes?

• What is the Rayleigh limit of doubly charged noble gas clusters?

• The size distribution of fission products.

• Why does the presently adopted approach result in much smaller appearance sizes than observed previously?

• Other issues: shell effects, doubly charged dimers and trimers.

It cannot be overemphasized that appearance sizes are purely experimental parameters. Initially, the appearance size (commonly called critical size in early reports) of a doubly charged cluster was supposed to mark the size where the repulsive Coulomb energy of the two separated holes in the cluster exceeds the binding energy of the cluster atoms.^39,77,78^ Clusters larger than *n*_a_ were supposed to be thermodynamically stable; no reference was made to nuclear fission, fission barriers,^79^ nor to Rayleigh^’^s work.^1^ However, the observation of metastable fission of triply charged clusters suggested that fission of (triply charged) clusters near *n*_a_ is thermally activated and competes with evaporation of monomers.^4,16,34,40,41,44^

In most experiments, noble gas cluster ions are formed by ionization of neutral precursors. The nascent ions are boiling hot and form an evaporative ensemble; cluster ions observed on experimentally relevant time scales of micro- to milliseconds are the products of one or more evaporations.^80,81^ Noble gas clusters are especially prone to dissociation because the missing electrons will eventually localize on Ng_2_^+^ or Ng_3_^+^ whose formation releases more than 1 eV per ionic core, greatly exceeding the evaporation energy of the cluster.^82–84^

When a large doubly charged cluster evaporates monomers, it will eventually shrink to a size where the rate of fission exceeds that of evaporation, because the fission barrier will quickly decrease with decreasing size while the evaporation energy remains approximately constant. We will designate the size where the rates of fission and evaporation are equal with *n*_a0_. Extending the approximation further, *n*_a0_ marks the size where the fission barrier (*E*_bar_) and evaporation energy (*E*_vap_) are equal.

It is not possible to prepare dications much smaller than *n*_a0_ by starting with dications above *n*_a0_ and exciting them by, say, collisions. This is nicely seen in the CID experiments where excitation of different precursors (for example Xe_*n*_^2+^ with *n* = 43, 49, 55) leads to approximately the same minimum size of doubly charged fragments, see Table 1. One perplexing observation, though, is that CID of Xe_43_^2+^, which is smaller than the previously reported appearance size (*n*_a_ = 47), does evaporate atoms at all, producing dications as small as Xe_39_^2+^, see Fig. 5(d). At the lowest collision pressure, the Xe_42_^2+^ product ion peak is much stronger than the sum of all fission fragments. We will return to this puzzling observation further below.

Most of the theoretical work published so far has been aimed at computing appearance sizes rather than Rayleigh limits because only the former can be compared to experimental data. The link with experiment is obvious in molecular dynamics studies of Xe_*n*_^2+^ and Ar_*n*_^2+^ where lifetimes have been estimated.^47–49,85^ Gay and Berne find that the lifetime of Xe_51_^2+^ is ≈100 ps while that of Xe_55_^2+^ is ≈10 μs.^47^ Goldberg *et al.* have modelled Ar_*n*_^2+^ and find that the fission time increases dramatically above *n* = 90.^48^ In spite of the nice agreement with experimental appearance sizes (see Table 1) one should note a disconnect between theory and experiment: theory models the fate of a dication after vertical ionization of a neutral cluster, with various assumptions concerning the initial location of the two holes, hole hopping, and formation of ionic cores. These ions carry several eV excess energy. In contrast, although the minimum life time of ions that form distinct mass peaks in the mass spectrum is about 50 μs, these ions may be the cold fragments formed from highly excited precursor ions during a much longer time window.^76,86^

Comparison between experiment and theory is less obvious when the dication is modelled as a liquid drop. In the simplest approach the fission barrier is calculated within the framework of the contacting-sphere model; the transition state occurs when the two spherical fragment clusters are brought into contact.^16,45,51,87^ With this model one obtains the most favourable fission channel as a function of cluster size, and the kinetic energy released upon fission. The size where the barrier vanishes for the most favourable channel is usually found to closely agree with the experimental appearance size, although one would rather expect it to agree with the (unknown) Rayleigh limit.

A more refined liquid drop model has been presented for Xe_*n*_^2+^ by Casero *et al.*^41^ The energy is computed for a deformable droplet as a function of its elongation. If the potential energy of the nascent (spherical) dication relative to the peanut-shaped equilibrium configuration exceeds the fission barrier, the droplet will undergo immediate fission within one vibrational period. If that first attempt at fission fails, the energy in the stretching mode, together with the energy released upon formation of the ionic cores, will dissipate into thermal energy which may or may not drive fission on a much slower time scale. Thus one has to consider two appearance sizes; one relates to “immediate” fission driven by the initial separation between the two charges, the other one to a thermally driven process. If the appearance size related to “thermal” fission (designated *n*_a0_ further above) is smaller than that related to “immediate” fission, the latter process will dictate the appearance size *n*_a_ in conventionally measured mass spectra, but collisionally excited dications will evaporate atoms (as seen in Fig. 5(d)) until their size drops well below *n*_a_.

Calculating the Rayleigh limit of noble gas clusters is not as simple as suggested by eqn (3). First, the surface tension *γ* depends on the temperature; for solid Ar at 0 K it is about 50% larger than for liquid Ar at 40 K.^51,61^ Second, *γ* depends on the droplet size.^60^ Third, Rayleigh's relation (eqn (3)) holds for a uniformly charged droplet which remains spherical up to the point where the fission barrier vanishes. In contrast, in its local energy minimum a droplet with two localized charges resembles a peanut.^41^

With these caveats in mind, we compute the Rayleigh limit from eqn (3) for two different temperatures and due consideration of the size dependence of the surface tension. The parameters for the surface tension of small liquid or solid argon clusters are taken from the molecular dynamics study of Briant and Burton.^61^ For Kr and Xe these parameters are assumed to scale with the energy parameter of the Lennard Jones potential.^16,51^ The results are listed in the last column of Table 1. We obtain *n*_Ray_ = 15 and 21 for solid and liquid Xe clusters, respectively. They are smaller, as they must, than the smallest Xe_*n*_^2+^ observed in the present work, *n*_a_ = 25. The fissility of Xe_25_^2+^ is *X* = 0.84 if referenced to the liquid phase. Similarly large fissilities have rarely been achieved, and so far only for alkali clusters.^13,53^ More commonly, the fissilities of the smallest observed dications are about 0.3 to 0.4.^4,13^

We now turn to the size distribution of fission products. They can only be meaningfully evaluated at the lowest collision pressure because at high pressure multiple collisions will lead to fragmentation of fission products. Fig. 5 presents the size distributions for the smallest dicationic clusters, *i.e.* for those with the lowest fission barrier. The bimodal distribution observed for Ar_77_^2+^ is reminiscent of the double-humped asymmetric distributions frequently observed in nuclear fission.^88^ The asymmetry, *α* = 0.17 (Table 2) is small, though. Moreover, the average number of monomers that are evaporated upon fission, is less than 1. For Kr_77_^2+^ and Xe_43_^2+^ the distributions of fission fragments are even narrower and single-humped. Their asymmetry parameters are even smaller than for Ar while the number of evaporated monomers is also near or below 1. Recall that the entries in Table 2 for the asymmetry parameter and the number of monomers lost represent upper limits, as discussed above in the context of eqn (2a) and (2b).

There are few experiments on van der Waals or weakly bound molecular clusters with which we can compare our results. Stace and coworkers have investigated metastable and collision-induced fragmentation of multiply charged van der Waals clusters. They observed evaporation from Ar_*n*_^2+^ but not its fission.^33,34^ In subsequent work they investigated dissociation of (NH_3_)_*n*_^2+^ with *n* just above its appearance size *n*_a_ = 50.^19^ Fission was very asymmetric and accompanied by loss of a large (≈13) number of molecules, probably because multiple collisions at high energies produced highly excited cluster ions.

Theoretical work suggests that the size distributions will depend on the fissility of the system.^4,89^ In the deformable droplet model, Xe_*n*_^2+^ will prefer symmetric fission if *n* = 40 but highly asymmetric fission if *n* = 60.^41^ The molecular dynamics simulation of Xe_51_^2+^ by Gay and Berne shows a pair of products with *α* = 0.41 and no concomitant loss of atoms.^47^ In a molecular dynamics simulation of Ar_*n*_^2+^, by Goldberg *et al.*,^48^ fission of Ar_90_^2+^ was characterized by *α* = 0.35 while much smaller asymmetries of *α* ≈ 0.1 were found for Ar_80_^2+^, Ar_70_^2+^, and Ar_55_^2+^; the number of neutral atoms lost in most simulation runs was 1. The authors observed that, after fission, the fragments evaporated some number of neutral atoms, which is at variance with the small (<1) number of lost atoms observed in the present study. The difference is likely related to the fact that the molecular dynamics study by Goldberg *et al.* pertains to highly excited ions, immediately after vertical ionization of their neutral precursors.

Why do we observe doubly charged clusters that are much smaller than reported previously? Their fission barriers are small, much smaller than their evaporation energies. Hence, they must remain cold throughout their synthesis. In previous work we succeeded in forming multiply charged alkali clusters in He nanodroplets close to the Rayleigh limit.^53^ We tentatively attributed their detection to two factors: first, a slow-down of the fission rate because clusters embedded in a HND are solid rather than liquid. Second, their formation by sequential rather than one-step ionization.

Concerning the first factor, we note that Möller and coworkers have reported a dramatic difference in the fission pattern of solid xenon clusters as compared to liquid neon clusters for fissilities near *X* = 1.^8^ A slower rate of fission increases the efficiency of the helium matrix in removing excess energy and quenching fission. On the other hand, in previous work we observed that the presence of a helium matrix barely reduces the appearance size of doubly charged CO_2_ clusters if the conventional approach (electron ionization of doped, neutral HNDs) is adopted.^55^

Hence the second factor, sequential ionization, must play a crucial role, as already conjectured in our previous report on doubly charged alkali clusters which were formed by ionization of doped, neutral HNDs.^53^ In the present experiments, sequential ionization is guaranteed as seen by the absence of dications when the second ionizer is turned off, see Fig. 2(b) and 3(b). But how, exactly, do the incident electrons convert monocations into a dications?

Broadly speaking, there are three pathways by which an incident electron may ionize a doped HND. First, the electron may directly ionize the dopant. Second, the electron may form He^+^ which moves toward the dopant by resonant charge hopping and ionizes the dopant by charge transfer (or the charge may be trapped on He_2_^+^ which then migrates towards the dopant). Third, the electron may electronically excite the helium, and the dopant is ionized by an intracluster Penning mechanism.

The efficiency of process (1) is small compared to (2) and (3) because the numerous helium atoms surrounding the dopant have a much larger total cross section than the embedded cluster.^90^

Charge transfer (process (2)) dominates if the dopant is neutral and heliophilic, but it is blocked by Coulomb repulsion if the dopant is positively charged. This leaves us with Penning ionization (process 3), which is efficient if the dopant resides on the surface because He* and 
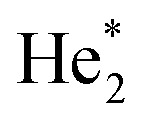
 are heliophobic, too.^56,67,91^ In a multiply charged droplet, the dopant ions reside close to the surface because of Coulomb repulsion, but they will be surrounded by one or a few solvation layers.^92^ Hence, it is not clear if heliophobic He* or 
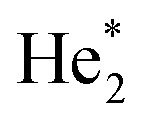
 will be able to Penning ionize the dopant ions.

However, photoelectron experiments of HNDs doped with noble gas atoms (which are heliophilic) suggest that Penning ionization does occur if the incident radiation leads to excitation of the 1s2p or higher bands.^93^ Furthermore, the seemingly low efficiency with which heliophilic dopants are Penning ionized may be due to the fact that Penning ions formed inside the HND tend to remain bound even if the Penning electrons are emitted.^57^

In closing, we mention two other topics. First, the height of the fission barrier *E*_bar_ is usually taken to be a continuous function of the cluster size but geometric shell effects may lead to oscillations in *E*_bar_(*n*).^51^ The oscillations may be caused by anomalies in the energy of the doubly charged precursor cluster or the energy of the fission fragments. The stability pattern of singly charged noble gas clusters is well known and easily seen, for example, in Fig. 5(d) where the “magic” Xe_19_^+^ and Xe_25_^+^ fragment ions appear with enhanced abundance. These anomalies correlate with anomalies in the evaporation energies of singly charged noble gas cluster ions. Likewise, anomalies in the size dependence of the fission barrier may produce anomalies in the abundance distribution of dications.^94,95^

One such example could be Kr_61_^2+^; its yield in Fig. 2(a) is large compared to its neighbours with half-integer *n*/*z* values. We have analyzed the spectrum with the software package IsotopeFit^70^ and determined the yield of Kr_*n*_^2+^ for odd and even values of *n*; results are presented in the ESI† (Fig. S6). Kr_61_^2+^ is, indeed, much more abundant than Kr_60_^2+^ or Kr_62_^2+^ but we cannot offer a compelling explanation for this anomaly. Smaller anomalies exist at Kr_53_^2+^ and Kr_55_^2+^ but their statistical significance is questionable.

Xe_43_^2+^ stands out in the mass spectrum of Xe clusters (Fig. 3(a)). It is tempting to attribute this anomaly to the fact that the next larger dication, Xe_44_^2+^, may produce two magic fragments upon fission, Xe_19_^+^ and Xe_25_^+^. Such a double magic decay would be accompanied by a reduced fission barrier. Additional CID experiments will be needed to test this conjecture.

Finally, contrary to what we have insinuated so far, doubly charged noble gas clusters well below the Rayleigh barrier may be metastable. In recent work, using the same experimental approach as discussed here, we have observed long-lived dicationic dimers and trimers of Kr and Xe.^59^ The fission barriers of these dimers, calculated with multi-reference methods such as MRCI, amount to a few hundred meV. They are not commonly observed because vertical ionization of neutral dimers will end in the repulsive part of the potential energy curves of Ng_2_^2+^.

## 5 Conclusions

Doubly charged clusters of argon, krypton and xenon that are much smaller than previously reported have been observed in mass spectra. For Kr and Xe, their +2 charge state has been confirmed by resolving their isotopologues. The clusters are smaller than deemed possible in some previous theoretical work; their sizes approach the Rayleigh limit. They have been prepared by first forming singly charged dopant clusters in helium nanodroplets and, in a separate step, exposing the doped HNDs to another electron beam. We propose that electronically excited He atoms convert the cold, solid dopant ions to dications by Penning ionization. For a better understanding of the mechanism by which dications are formed it would be useful to measure the ion yield *versus* the energy of the incident electrons, but this kind of experiment requires higher ion yields than attainable right now. Also useful will be a comparison of data obtained with the current approach, and with the more common approach, *i.e.* electron ionization of doped, neutral HNDs. These experiments are currently underway in our lab for HNDs doped with CO_2_.

Experiments with collisionally excited dications reveal the following: (i) when the collision gas pressure is at a minimum and the size of the precursor ions is smaller than previously reported appearance sizes, the size distributions of the fission fragments are very narrow, *i.e.* characterized by small asymmetry parameters. Furthermore, the distribution of fragments is nearly symmetric with respect to the precursor ion, *i.e.* fission is accompanied by evaporation of no more than ≈1 atom. (ii) Larger precursor ions evaporate several atoms upon mild collisional excitation; the doubly charged fragments will not undergo fission until their size has decreased below previously reported appearance sizes.

Hopefully, our experimental results will stimulate theoretical work. So far only one report has addressed the value of the Rayleigh limit.^41^ The result, *n*_Ray_ = 30, exceeds the size of the smallest observed dications observed here, Xe_25_^2+^. The discrepancy may be rooted in deficiencies of the model, or in uncertainties of the surface tension *γ*. The large dependence of *γ* on the temperature and phase of the system (liquid *versus* solid) leads to questionable results if they are based on liquid droplet models. Molecular dynamics simulations that attempt to minimize the energy of a doubly charged cluster as a function of the separation between the two charges are probably more suited to determine the Rayleigh limit. The low value of the Rayleigh limit for Xe clusters, *n*_Ray_ < 25, should make molecular dynamics simulations feasible. Still, the extent to which the electron holes are distributed may have a noticeable effect on cluster stability; this will be a challenge to theory.^96,97^

## Data availability

The data that support the findings of this study are available from the corresponding authors upon reasonable request.

## Author contributions

Conceptualization, E. G. (E. Gruber) and P. S. (P. Scheier); formal analysis, E. G., I. S. (I. Stromberg), S. B. (S. Bergmeister), and O. E. (O. Echt); funding acquisition, P. S.; investigation, E. G., I. S., S. B., and L. G. (Lisa Ganner); methodology, E. G. and P. S.; project administration, P. S.; resources, F. Z. (Fabio Zappa) and P. S.; supervision, E. G. and P. S.; visualization, E. G., I. S., and O. E.; writing – original draft, O.E.; writing – review & editing, E. G., I. S., S. B., P. S. and O. E. All authors have read and agreed to the published version of the manuscript.

## Conflicts of interest

We declare no conflict of interest.

## Supplementary Material

CP-026-D4CP00658E-s001
