# Supplementary material for: Size limits and fission channels of doubly charged noble gas clusters
Source: Phys Chem Chem Phys. 2024 Mar 26;26(18):13923–36. doi: 10.1039/d4cp00658e (PMC11078200; doi:10.1039/d4cp00658e)
Supplement: CP-026-D4CP00658E-s001 [file CP-026-D4CP00658E-s001.pdf]

Electronic Supplementary Material (ESI) for Physical Chemistry Chemical Physics.
This journal is © the Owner Societies 2024

Electronic supplementary information (ESI)

Size Limits and Fission Channels of Doubly Charged Noble Gas Clusters

Ianessa Stromberg,[1,2] Stefan Bergmeister,[1] Lisa Ganner,[1], Fabio Zappa,[1] Paul Scheier,[1] Olof Echt,[1,3*] Elisabeth Gruber[1*]

1 Institut für Ionenphysik und Angewandte Physik, Universität Innsbruck, Innsbruck, Austria
2 School of Chemistry, University of Edinburgh, Edinburgh, United Kingdom
3 Department of Physics, University of New Hampshire, Durham, USA

* Corresponding authors. E-mail address e.gruber@uibk.ac.at (E.G.), olof.echt@unh.edu (O.E.)

List of material

Fig. S1 Schematic of the experimental apparatus.
Fig. S2 Sections of mass spectra of HNDs doped with Kr.
Fig. S3 Mass spectrum of Ar clusters, and comparison with expected isotope patterns.
Fig. S4 CID mass spectra of Ar_{115}^{2+}.
Fig. S5 CID mass spectra of Xe_{55}^{2+}
Fig. S6 Abundance of Kr_n^+ and Kr_n^{2+} *versus* size n.
Table S1 The minimum distance between charge carriers in multiply charged HNDs.

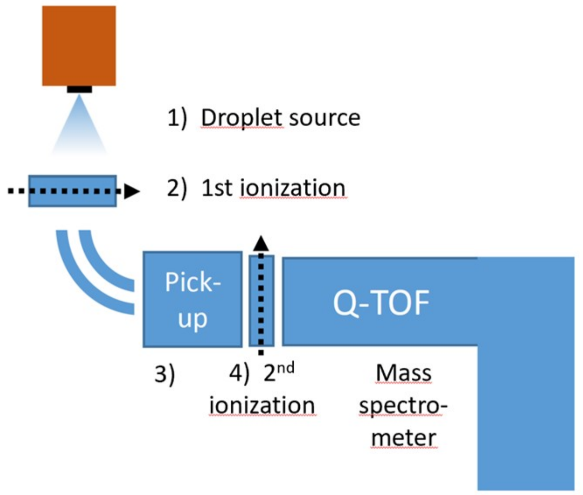

Fig. S1
Extraction of dopant ions emerging from a collision cell filled with He and collision-induced dissociation of mass selected ions occur in the Q-TOF which is a modified commercial Quadrupole mass spectrometer (Q-TOF Ultima) from Micromass, Waters).

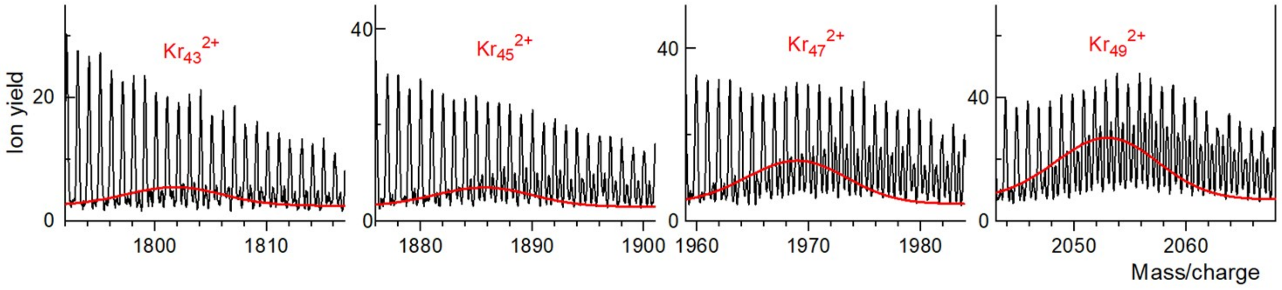

Fig. S2
Sections of a mass spectrum of HNDs doped with Kr. Red lines indicate the expected distributions of isotopologues of Kr_n^{2+}. Kr_{43}^{2+} is the smallest doubly charged cluster that can be identified.

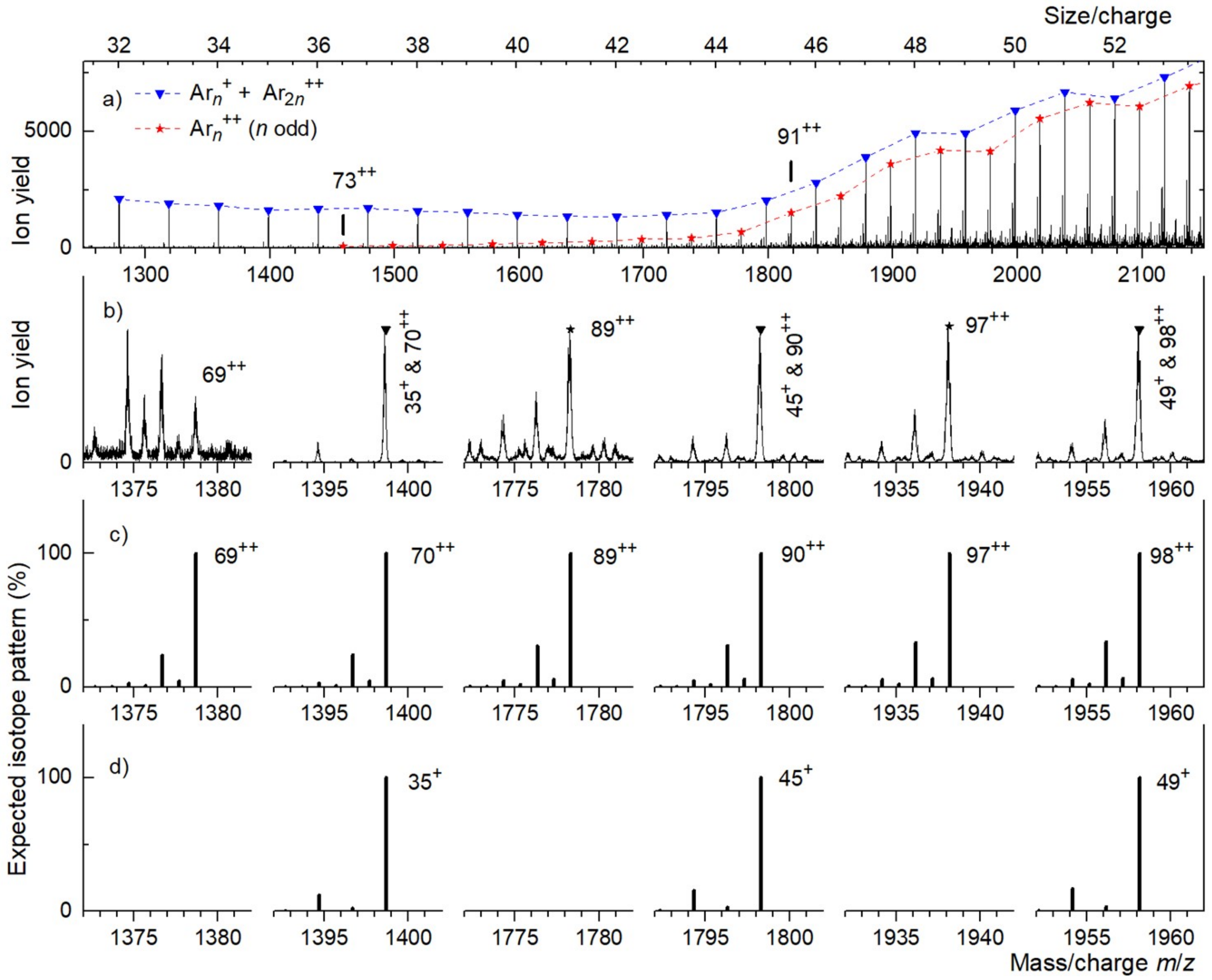

Fig. S3

Panel a: A mass spectrum of HNDs doped with Ar. The prominent series of mass peaks are due to Ar_n^{2+} (n odd) and Ar_n^+ combined with Ar_{2n}^{2+}; they are marked by asterisks and triangles, respectively. Panel b zooms into sections of the spectrum where Ar_n^{2+} with $n = 69$, 70, 89, 90, 97, and 98 would show up; singly charged cluster ions with $n = 35$, 45, and 49 would contribute to three of these sections. Mass peaks that arise from isotopically pure $^{40}Ar_n^{z+}$ are marked by triangles and asterisks for integer and half-integer values of n/z, respectively. The size n and charge state z are indicated by n^{z+}.

Cluster ions with $n/z = 49$ would show up in the right-most section in panel b. The expected pattern of isotopes expected for this region is displayed below in panel c for 98^{++}, and further below in panel d for 49^+. Their isotope patterns are markedly different. The expected isotope pattern of 98^{++} matches the experimental data while that of 49^+ does not. This proves the assertion that the main contribution to the peak at $n/z = 49$ comes from dications rather than monocations.

The two minor mass peaks in the mass region due to $n/z = 45$ ions are about equally high; monocationic and dicationic clusters contribute about equally. In the spectrum at $n/z = 44.5$ the mass peaks at 2 and 4 units below the main peak are more intense than expected for 89^{++}, and additional mass peaks appear. Ions other than pure Ar_n^{2+} contribute in this section. The mass spectrum at $n/z = 35$ is well accounted for by the expected isotope pattern of 35^+, but for $n/z = 34.5$ the isotope pattern of Ar clusters does not account for the measurement which is swamped by ions that contain impurities, such as $H_2OAr_n^{z+}$, or by $He_pAr_n^{z+}$.

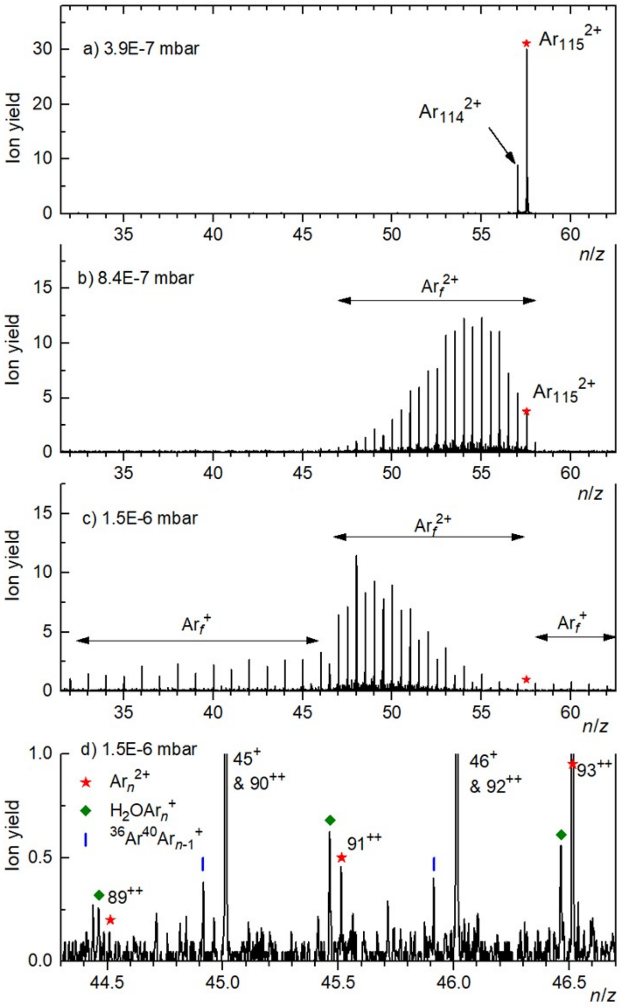

Fig. S4

Panels a through c: Fragment ions produced from Ar_{115}^{2+} precursor ions by collisions with argon gas. The collision gas pressure is increasing from top to bottom; values are indicated. The charge state of the dominant fragment ions is indicated above the horizontal arrows. Panel d zooms into a section of the spectrum displayed in panel c.

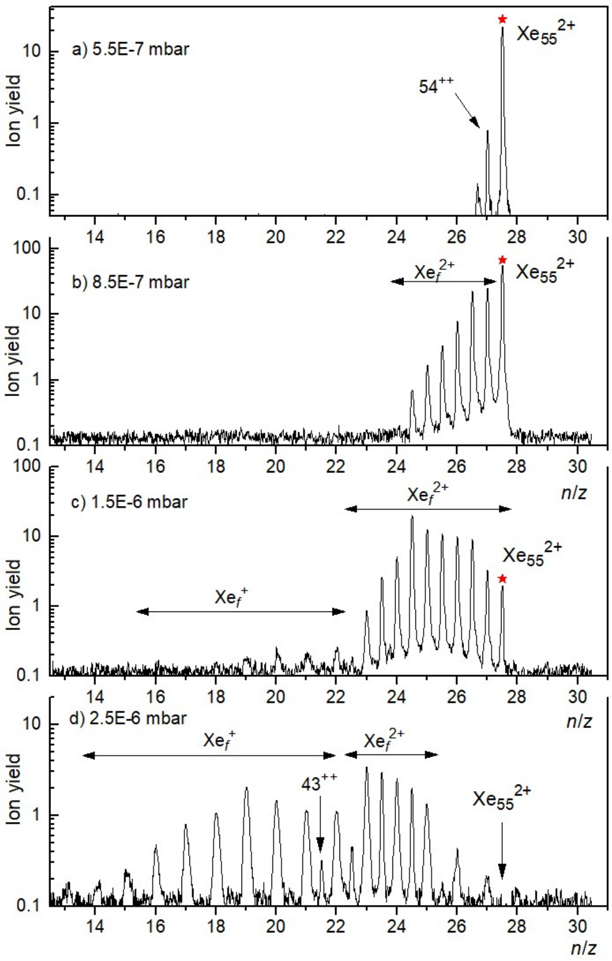

Fig. S5
Fragment ions produced from precursor ions Xe_{55}^{2+} by collisions with argon gas. The collision gas pressure is increasing from top to bottom; values are indicated. The charge state of the dominant fragment ions is indicated above the horizontal arrows.

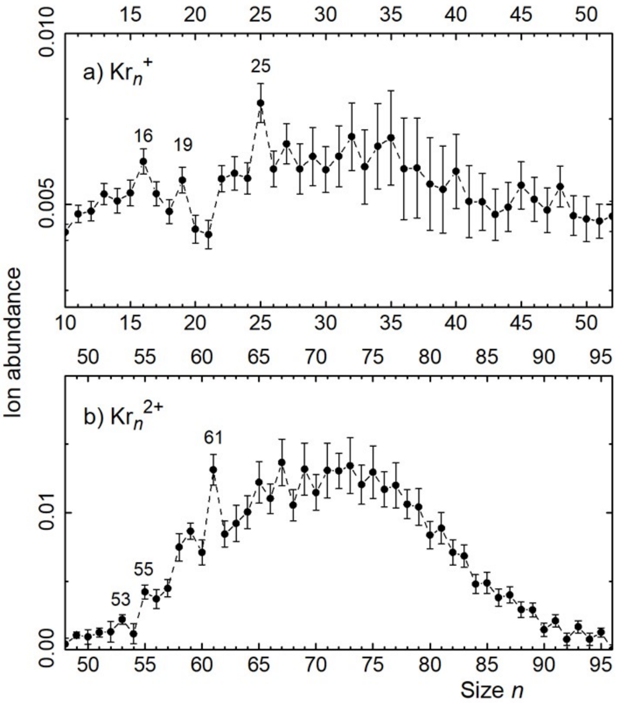

Fig. S6
Abundance of singly and doubly charged Kr clusters. The abundance of dications with integer values of n/z has been deduced from the mass spectrum with the fitting routine IsotopeFit.

Table S1 The minimum distance between charge carriers in multiply charged HNDs

Based on the assumption that the charges in a spherical multiply charged HND arrange according to the Thomson problem (https://doi.org/10.1080/14786440409463107), we calculate the minimum distance between two charge carriers by multiplying the shortest distance taken from Wales and Ulker (https://www-wales.ch.cam.ac.uk/~wales/CCD/Thomson/table.html) with the radius of the HND. With the critical sizes published by Laimer et al. (https://doi.org/10.1103/PhysRevLett.123.165301) we obtain the following values of d_{min} for z=2 up to 55.

Charge state z	Critical size n_c	d_{min} (nm)
2	99543	20.58
3	162889	21.00
4	217186	21.79
5	271482	20.33
6	334828	21.80
7	411748	19.41
8	552013	22.14
9	626671	21.19
10	733001	21.51
21	2212578	21.82
23	2669573	21.81
35	4249252	20.48
44	6131064	21.89
51	7739709	21.37
55	9348355	21.53
